# Cognitive Impairment in Parkinson’s Disease Is Reflected with Gradual Decrease of EEG Delta Responses during Auditory Discrimination

**DOI:** 10.3389/fpsyg.2018.00170

**Published:** 2018-02-21

**Authors:** Bahar Güntekin, Lütfü Hanoğlu, Dilan Güner, Nesrin H. Yılmaz, Fadime Çadırcı, Nagihan Mantar, Tuba Aktürk, Derya D. Emek-Savaş, Fahriye F. Özer, Görsev Yener, Erol Başar

**Affiliations:** ^1^Department of Biophysics, School of International Medicine, Istanbul Medipol University, Istanbul, Turkey; ^2^REMER, Clinical Electrophysiology, Neuroimaging and Neuromodulation Lab, Istanbul Medipol University, Istanbul, Turkey; ^3^Department of Neurology, School of Medicine, Istanbul Medipol University, Istanbul, Turkey; ^4^Department of Neuroscience, Aziz Sancar Institute of Experimental Medicine Research, Istanbul University, Istanbul, Turkey; ^5^Department of Neuroscience, Institute of Medical Science, Istanbul Medipol University, Istanbul, Turkey; ^6^Department of Psychology, Faculty of Letters, Dokuz Eylül University, Izmir, Turkey; ^7^Department of Neurology, Koç University Hospital, Istanbul, Turkey; ^8^Department of Neurology, Dokuz Eylül University School of Medicine, Izmir, Turkey; ^9^Brain Dynamics Multidisciplinary Research Center, Dokuz Eylül University, Izmir, Turkey; ^10^Izmir International Biomedicine and Genome Institute, Dokuz Eylül University, Izmir, Turkey; ^11^Brain Dynamics, Cognition and Complex Systems Research Center, Istanbul Kultur University, Istanbul, Turkey

**Keywords:** Parkinson’s disease, EEG, event related oscillations, delta, oddball paradigm

## Abstract

Parkinson’s disease (PD) is a neurodegenerative disease that is characterized by loss of dopaminergic neurons in the substantia nigra. Mild Cognitive impairment (MCI) and dementia may come along with the disease. New indicators are necessary for detecting patients that are likely to develop dementia. Electroencephalogram (EEG) Delta responses are one of the essential electrophysiological indicators that could show the cognitive decline. Many research in literature showed an increase of delta responses with the increased cognitive load. Furthermore, delta responses were decreased in MCI and Alzheimer disease in comparison to healthy controls during cognitive paradigms. There was no previous study that analyzed the delta responses in PD patients with cognitive deficits. The present study aims to fulfill this important gap. 32 patients with Parkinson’s disease (12 of them were without any cognitive deficits, 10 of them were PD with MCI, and 10 of them were PD with dementia) and 16 healthy subjects were included in the study. Auditory simple stimuli and Auditory Oddball Paradigms were applied. The maximum amplitudes of each subject’s delta response (0.5–3.5 Hz) in 0–600 ms were measured for each electrode and for each stimulation. There was a significant stimulation × group effect [*F*_(df = 6,88)_ = 3,21; *p* < 0.015; $\eta_{p}^{2}$ = 0.180], which showed that the difference between groups was specific to the stimulation. Patients with Parkinson’s disease (including PD without cognitive deficit, PD with MCI, and PD with dementia) had reduced delta responses than healthy controls upon presentation of target stimulation (*p* < 0.05, for all comparisons). On the other hand, this was not the case for non-target and simple auditory stimulation. Furthermore, delta responses gradually decrease according to the cognitive impairment in patients with PD.

**Conclusion**: The results of the present study showed that cognitive decline in PD could be represented with decreased event related delta responses during cognitive stimulations. Furthermore, the present study once more strengthens the hypothesis that decrease of delta oscillatory responses could be the candidate of a general electrophysiological indicator for cognitive impairment.

## Introduction

Parkinson’s disease (PD) is a neurodegenerative disease that is characterized by loss of dopaminergic neurons in the substantia nigra. Primary symptoms of the disease include motor symptoms like tremor, rigidity, postural instability, bradykinesia. Motor symptoms show a good response to levodopa. Disorders of cognitive functions, such as impairments in executive functioning, working memory and attention may also be present in PD (Soliveri et al., 2000; Lewis et al., 2003; Pollux, 2004). Mild cognitive impairment (MCI) in PD was first described by Caviness et al. (2007a) as an intermediate condition between normal cognition and dementia (Aarsland et al., 2011). Studies show that the clinical picture that starts as MCI progresses to dementia in 60% of the patients (Hughes et al., 2000; Levy et al., 2000; Aarsland et al., 2003; Buter et al., 2008). Recent evidence suggests there may be subtypes of PD that may affect neurotransmitter systems other than dopamine, manifesting with different cognitive/behavioral courses (Kehagia et al., 2010; Bohnen and Albin, 2011; Moustafa and Poletti, 2013).

Today, motor symptoms of PD have become modifiable to a great extent with treatment strategies, which resulted in an increasing interest in the “non-motor” components of the disease, particularly cognitive decline, and dementia. Cross-sectional studies report the frequency of dementia in PD as 30%, whereas follow-up studies show very high rates (up to 80%) in the long-term (Lin and Wu, 2015). Beyond other non-motor symptoms, PD-dementia (PDD) is the most important determinant of mortality, patient care, and life quality. PD shortens patient’s life expectancy, and currently, no effective treatment method exists (Levy et al., 2002; Kehagia et al., 2010). For these reasons, detecting patients that are likely to develop dementia, that is, identification of new indicators is of significance with regard to bringing up possible treatment options.

Electroencephalogram (EEG) research on Parkinson’s disease was mostly performed with analysis of Spontaneous EEG and/or event related potentials. In the spontaneous EEG analysis, the researchers indicated slowing of delta and theta activity in PD patients in comparison to healthy controls (Neufeld et al., 1988, 1994; Bonanni et al., 2008; Serizawa et al., 2008; Pugnetti et al., 2010). Increased delta and theta and reduced alpha and beta activity were also reported for PD patients with dementia (Caviness et al., 2007b; Babiloni et al., 2017). Event related potentials of PD patients were also investigated upon application of several paradigms.

Authors investigated ERP components both during visual and auditory stimulations. In a recent review (Seer et al., 2016) indicated that there were 65 different studies investigating the P3b in patients with PD. These authors further reported that in 19% of these studies showed reduced P3b amplitude in PD patients in comparison to healthy controls, 9% of these studied found increased P3b amplitudes in PD patients and finally 72% reported no differences between healthy controls and PD patients (Seer et al., 2016). On the other hand, P3 latency was reported to be prolonged in PD patients in comparison to healthy controls, and this prolongation was mainly found in PD patients with dementia. To our knowledge, the first paper on Event Related Potentials in PD was published by (Hansch et al., 1982). These authors analyzed visual and auditory event related potentials in PD and reported increased latencies for both P200 and P300 components of auditory ERP. These authors also reported reduced P300 amplitudes in PD patients during visual paradigm. Studies on P3b amplitude in PD showed contradictory results during auditory paradigms. Some researchers showed decreased P3b responses in PD (Gil et al., 1989; Hautecoeur et al., 1991; Aotsuka et al., 1996; Philipova et al., 1997; Jiang et al., 2000) while the others reported increased responses (Green et al., 1996; Tanaka et al., 2000). Most of the other studies did not find significant results between subject groups (O’Donnell et al., 1987; Chia et al., 1995; Kim et al., 1995; Elwan et al., 1996, Please see Seer et al., 2016 for more detail information).

P300 components in the ERP is one of the most important potentials analyzed in the investigation of cognitive functions of several patient groups. As the P300 component has amplitude and time characteristics, it also has frequency characteristics. Researchers showed that mainly delta, theta, alpha, and gamma frequency bands were involved in P300 potential (Başar-Eroglu et al., 1991, 1993, 2000; Intriligator and Polich, 1994; Kolev et al., 1997; Schurmann et al., 1997; Demiralp et al., 1999, 2001; Spencer and Polich, 1999; Karakaş et al., 2000; Yordanova et al., 2000, 2001; Sakowitz et al., 2001; Güntekin et al., 2013). Accordingly, it is also essential to analyze the frequency properties of event related potentials using event related oscillatory methodologies.

Delta oscillatory responses merit special attention in the study of cognitive impairment. Event related delta responses were mainly correlated with attention, perception and decision making processes (see reviews Knyazev, 2012; Güntekin and Başar, 2016). Delta responses increase during cognitive load mainly in frontal, central and parietal locations. Increase of delta response during target detection in both visual and auditory paradigms were reported several times (Başar et al., 1984; Başar-Eroglu et al., 1992; Kolev et al., 1997; Demiralp et al., 1999, 2001; Bernat et al., 2007; Mathes et al., 2012; Prada et al., 2014). On the other hand when the stimulation was an emotional paradigm, face or face expression paradigm then the delta responses were increased mainly at parietal and occipital locations (Güntekin and Başar, 2014).

As we have also indicated in our recent review article, decrease of delta responses appear to be a general electrophysiological indicator in search of cognitive impairment (Güntekin and Başar, 2016). The literature showed that delta responses decreased in Alzheimer’s disease patients, in MCI, in bipolar disorder and as well in schizophrenia (Ergen et al., 2008; Ford et al., 2008; Yener et al., 2008, 2012, 2013; Atagün et al., 2014; Kurt et al., 2014). There are few studies on the event related oscillatory responses of patients with Parkinson’s disease. Ellfolk et al. (2006) reported that event related alpha synchronization in the posterior electrodes was observed in the control group but not in the PD patients during auditory-verbal memory task. Schmiedt et al. (2005) indicated that PD patients had less theta increase and upper alpha suppression than healthy controls during visual discrimination performance. Dushanova et al. (2010) showed that healthy controls had a higher event related beta synchronization than PD patients in a late time window. These authors also found differences between groups in different time and frequency bands of gamma activity. Schmiedt-Fehr et al. (2007) reported increased delta responses over parietal and occipital electrodes during Simon task. In a recent study with a different group of subjects, we have shown that delta responses also reduced in PD patients without cognitive deficits during a visual oddball paradigm (Emek-Savaş et al., 2017). However, the change of delta responses in PD patients with cognitive impairment is still unknown. Furthermore, there were no previous studies analyzing event related delta responses during auditory oddball paradigm. The present study aims to fulfill these important gaps. In the present study different group of PD patients were included in the experiments to see how delta responses would change in PD patients with mild cognitive deficits and in PD patients with dementia. We hypothesize that as the cognitive functions decline in PD patients, delta responses will reduce more. In accordance with this view, the patients with dementia would have more reduced delta responses than the healthy controls, and PD patients with MCI, or PD patients without cognitive deficits.

## Materials and Methods

### Patient Selection and Clinical Evaluation

Patients included in this study were the ones who referred to Movement Disorders clinics at Istanbul Medipol University Hospital and who approved participation in the study. The diagnosis of PD was based on the criteria of “United Kingdom Parkinson’s Disease Society Brain Bank” (Daniel and Lees, 1993). Again in this frame; patients who had previously suffered head trauma, stroke, who had been exposed to the toxic substance, who implied Parkinson plus syndromes in neurological examinations and patients with pyramidal, cerebellar examination findings, gaze paresis and autonomic dysfunction were excluded from the study. The ethical committee of Istanbul Medipol University (No: 10840098-51) approved the study. Informed consent was obtained from all participants or caregivers.

The Unified Parkinson’s Disease Rating Scale (UPDRS) (Lang and Fahn, 1989) was used in order to determine the clinical features of PD; and the Hoehn-Yahr scale (Hoehn and Yahr, 1967) was used to determine the disease stage. Drug treatments related to the disease were not intervened, and the total daily doses of dopa and equivalent dopa agonist doses were calculated as proposed by Fénelon et al. (2000).

All patients with PD were evaluated 60–90 min after their morning dose of levodopa for the EEG recordings.

**FIGURE 1:**
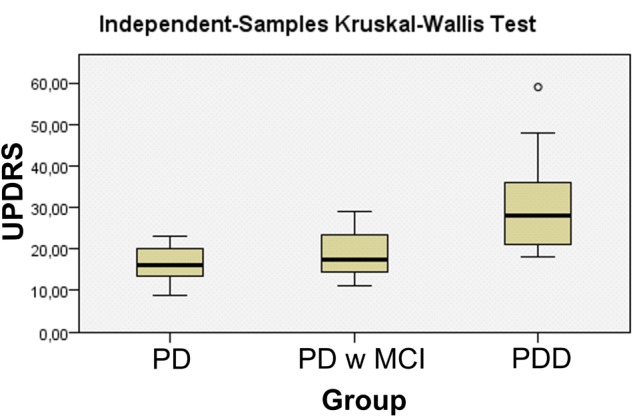
Unified Parkinson’s Disease Rating Scale (UPDRS) scores of Patients with dementia Parkinson’s disease.

**FIGURE 2:**
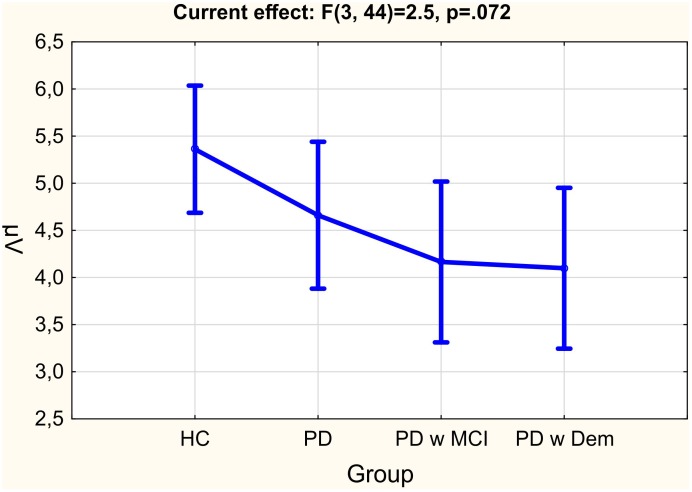
The mean values of delta responses for all group of subjects; healthy controls (HC), Parkinson’s disease without cognitive deficit (PD), Parkinson’s disease with MCI (PD w MCI), and Parkinson’s disease with dementia (PD w Dem).

Twelve Parkinson patients without cognitive deficits (*M* = 61.75, *SD* = 6.09), 10 PD patients with MCI (*M* = 66.1, *SD* = 7.12), 10 PD patients with dementia (*M* = 68.4, *SD* = 7.32) and 16 healthy elderly controls (*M* = 61.06, *SD* = 7.24) were included in the study. **Table 1** represents the demographic information of the subject groups. The standardized Mini-mental Examination State (MMSE) test was significantly different between groups. The healthy group had significantly higher MMSE scores (*M* = 27.67, *SD* = 1.44) than all PD groups, namely, PD without cognitive deficit (*M* = 26.33, *SD* = 1.99), PD with MCI (*M* = 24.1, *SD* = 2.47), and PD with dementia (*M* = 18.4, *SD* = 3.98) (**Table 1** and **Figures 1**, **2**). UPDRS scores of PD with dementia (*M* = 31.90, *SD* = 12.97) were higher than PD with MCI (*M* = 18.78, *SD* = 6.17) and PD without cognitive deficits (*M* = 16.54, *SD* = 4.54). This distribution of UPDRS scores is not the same across the groups (*p* = 0.003). *Post hoc* analysis shows that this group difference mostly represents to the difference between PD with dementia and PD without cognitive deficits (*p* = 0.006) (**Table 1** and **Figure 1**).

**Table 1:** Demographics and scores of the Unified Parkinson’s Disease Rating Scale (UPDRS) (motor) and the standardized Mini-Mental Examination Test in healthy controls and all groups of Parkinson’s disease.

	HC (*N* = 16)	PD (*N* = 12)	MCI (*N* = 10)	PDD (*N* = 10)	
	*M* ±*SD*	*M* ±*SD*	*M* ±*SD*	*M* ±*SD*	*p*
Age	61.06 ± 7.24	61.75 ± 6.09	66.1 ± 7.12	68.4 ± 7.32	0.072^a^
Gender (M/F)	7/9	8/4	8/2	9/1	0.071^b^
UPDRS (Motor)	–	16.54 ± 4.54	18.78 ± 6.17	31.90 ± 12.97	0.003^a^
MMSE	27.67 ± 1.44	26.33 ± 1.92	24.1 ± 2.47	18.4 ± 3.98	0.000^b^

*M, mean; SD, standard deviation; HC, healthy controls; PD, Parkinson’s disease without cognitive deficits; PD MCI, Parkinson’s disease with mild cognitive impairment; PDD, Parkinson’s disease with dementia; M, Male; F, Female; MMSE, The Standardized Mini Mental Examination Test, ^*a*^Kruskal–Wallis-h, ^*b*^Chi Square Test.*

### Behavioral and Neuropsychometric Evaluation

Standardized Mini Mental Test for General Cognitive Assessment (MMSE) (Gungen et al., 2002), verbal memory processes test (SBST) (Öktem, 2011) and visual subtest of Wechsler Memory Scale (Wechsler, 1987) for the evaluation of memory functions, Stroop Color Word Test (Karkas, 2006), Clock Drawing Test (Brodaty and Moore, 1997) and Categorical Verbal Fluency Test (Crawford, 1992) for the evaluation of administrative functions, Turkish versions of Benton’s Face Recognition Test (BFR) and Benton Line Judgment Orientation Test (BLOT) (Karkas, 2006) for the evaluation of visuospatial functions were used.

Grading of the cognitive status of patients and MCI diagnosis were performed by the applied neuropsychometric tests, in the framework of criteria defined by Litvan et al. (2012). Again for the diagnosis of dementia, the criteria for dementia in PD defined by Emre et al. (2007) were used. Staging of the dementia was performed using the Clinical Dementia Rating Scale (CDR) (Morris, 1993).

The control group consisted of subjects of similar age, gender, and education level. The control group was formed from patient relatives who were practically informed and gave approval. The control group was also neurologically evaluated by a neurologist specialized in movement disorders (LH, NY). Those having exposure to neurological disease history like toxic substances, head trauma, stroke or those diagnosed with dementia and those with findings as evidence of cognitive impairment and dementia during the neuropsychometric evaluation, were excluded from the study. The protocol applied to PD patients were also applied to the control group.

### Procedure and Stimuli

Two types of stimuli were presented: simple auditory stimuli and auditory oddball paradigm. The auditory stimuli had a 1,000 ms duration and were presented by two loudspeakers. The auditory simple stimuli were tones of 80 dB and 1,500-Hz tones. The inter-stimulus intervals varied randomly between 3 and 7 s. The total number of stimuli was 60. A classical auditory oddball paradigm was used in the experiments. Two types of stimuli were used: task relevant target and task- irrelevant non-target (standard). The total number of stimuli was 120 (40 target, 80 non-target). In the oddball paradigm, the 80 dB, 1600-Hz tones (target) and 1500 Hz tones (non-target) were presented in a random sequence. The interval between tones varied randomly between 3 and 7 s. The subjects were instructed to keep a mental count of the number of 1600-Hz tones (target).

### EEG Recordings

EEG of all subjects were recorded in a dimly lit and isolated room which was at the Istanbul Medipol University Hospital, REMER, Clinical Electrophysiology, Neuroimaging and Neuromodulation Laboratory. EEG was recorded from 32 Ag/AgCl electrodes according to the international 10–20 System with Brain Amp 32-channel DC system machine with band limits of 0.01–250 Hz and digitized on-line with a sampling rate of 500 Hz. Two earlobe electrodes (A1–A2) were served as reference electrodes. All impedances kept below 10 kΩ. EOG was recorded from medial upper- and lateral orbital rim of the right eye with Ag/AgCl electrodes.

### EEG Analysis

EEG data pre-processing and EEG analysis were performed by Brain Vision Analyzer 2 Software, F_3_, F_4_, C_3_, C_4_, T_7_, T_8_, TP_7_, TP_8_, P_3_, P_4_, O_1_, and O_2_ were analyzed. Before the analysis, the artifacts in the EEG were rejected off-line, EEG and EOG recordings were examined visually. Trials with muscle artifacts, eye movement, and blink artifacts were rejected. EEG was segmented for 1000 ms before and 1000 ms after stimulus. Epochs were than averaged to obtain Event Related Potentials for each stimulus, for each electrode, and for each subject. These ERPs were then digitally filtered in 0.5–3.5 Hz band limits for analyzing event related delta responses. After obtaining delta responses for each subject, for each stimulation and for each electrode grand averages were analyzed for each group. These grand averages included 16 subjects for healthy controls, 12 patients with Parkinson’s disease without cognitive deficits, 10 patients with Parkinson’s disease with MCI and 10 patients with Parkinson’s disease with dementia. A separate grand average including all Parkinson patients (N = 32) was also analyzed to observe the differences between the different group of subjects. After comparing grand averages, we have observed that there was a gradual decrease of delta responses from Parkinson’s disease without cognitive deficits to PD with MCI and to the PD dementia at the end. Accordingly, we have decided to analyze the delta responses of the patient groups by taking into consideration of their cognitive deficits. The epoch numbers of simple auditory stimulation, target, and non-target responses were equalized randomly.

The maximum peak to peak event related delta responses were measured for each stimulation, for each electrode and each subject in 0–600 ms. These data were then used for statistical analysis.

### Statistical Analysis

Statistical analysis was performed with Repeated Measures of ANOVA included in SPSS software. Three Stimulation (simple auditory, target, non-target) × six location (frontal, central, temporal, tempo-parietal, parietal, occipital) × two hemisphere (right, left) were included as within subjects; four groups (Healthy controls, Parkinson’s disease without cognitive deficit, PD with MCI, and PD with dementia) was included as between subject factor. Greenhouse–Geisser corrected p-values were reported. Post hoc analyses were performed by Bonferroni test with Statistica software. The comparisons of the neuropsychological tests between subject groups were performed by Kruskal–Wallis Test.

## Results

### Behavioral Results

In each measuring session, there were 40 target stimulations. Nine of the healthy control subjects counted the target stimulation as 40; three of the healthy subjects made one mistake while counting the target stimulation, and four of them made more than one mistake (minimum = 2, maximum = 5). Six of the Parkinson’s patients without cognitive deficits counted the target stimulation as 40; four of them made one mistake, and two of them made two mistakes. Three of the patients with PD-MCI counted the target stimulation as 40; four of the patients with PD-MCI made one mistake, and three of them made more than one, respectively, two mistakes, four mistakes and eight mistakes. Only one of the patients PD with dementia could counted the target stimulation as 40, and two of the patients with PD dementia made one mistake; and seven of them made more than six mistakes (minimum: 6, maximum: 40, mean: 23,57, SD: ± 11.21). We have performed correlation analysis between the number of mistakes that were made by the subjects and delta amplitudes for all electrodes analyzed. If the subject has counted the target as 46 or 34 in these both conditions the number of mistakes was defined as “six”. Kendall’s Correlation analysis showed that delta response were negatively correlated with the increasing number of mistakes at central locations [C3 (*p* = 0.022), Cz (*p* = 0.034), C4 (*p* = 0.006)]; right temporal locations [T8 (*p* = 0.03)]; temporo-parietal locations [Tp7 (*P* = 0.014), Tp8 (*p* = 0.001)] and parietal locations [P3 (*p* = 0.003), Pz (*p* = 0.004), P4 (*p* = 0.003)] upon presentation of target stimulation. As the number of mistakes increased delta responses decreased.

### Results of Delta Responses

**FIGURE 3:**
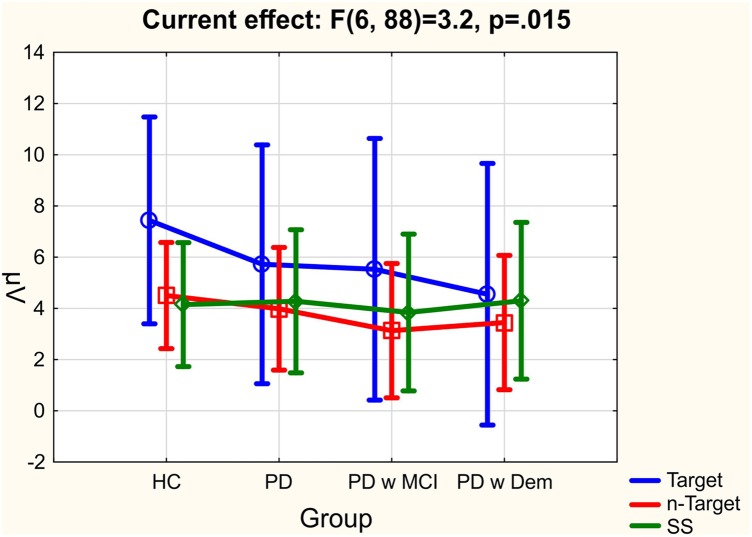
The mean values of delta responses upon presentation of target (blue line), non-target (red line) and simple auditory (green line) stimulation for all group of subjects; healthy controls (HC), Parkinson’s disease without cognitive deficit (PD), Parkinson’s disease with MCI (PD w MCI), and Parkinson’s disease with dementia (PD w Dem).

**Table 2** represents the significant results for all comparisons. Comparison between groups was near to the significant level [*F*_(df=3,44)_ = 2,5; *p* = 0.072; $\eta_{p}^{2}$ = 0.146]. Healthy controls had higher delta responses in comparison to all other patient groups. PD patients with dementia had the lowest delta response. **Figure 2** shows the mean values for group comparisons. The difference between groups was specific to the stimulation. There was a significant stimulation × group effect [*F*_(df=6,88)_ = 3,21; *p* = 0.015; $\eta_{p}^{2}$ = 0.180], patients with Parkinson’s disease (including PD without cognitive deficit, PD with MCI, and PD with dementia) had reduced delta responses than healthy controls upon presentation of target stimulation. *Post hoc* comparisons showed this difference was significant between healthy controls and PD patients with dementia (*p* = 0.003). On the other hand, this was not the case for non-target and simple auditory stimulation. The **Figure 3** and **Table 3** represents significant stimulation × group comparisons. The mean values of delta responses upon presentation of the target (blue line), non-target (red line) and simple auditory (green line) stimulation were presented in **Figure 3**. As it can be seen in the figure and the table the healthy controls had the highest delta responses upon target stimulation in comparison to non-target (*post hoc* comparisons *p* < 0.0001) and simple auditory stimulation (*post hoc* comparisons *p* < 0.0001). The difference between target stimulation vs. non-target and simple auditory stimulation is evident for healthy controls, but these differences are less apparent in PD patients with dementia. Furthermore, the group differences are clearly seen in response to target stimulation. Healthy controls had the highest and PD patients with dementia had the lowest delta responses in response to target stimulation [*post hoc* comparisons between HC and PD with dementia (*p* = 0.003)].

**Table 2:** Significant Comparisons between conditions.

Within-subjects effects	*F*	df	*P*	$\eta_{p}^{2}$
Stimulation	2,88 = 33,1	2	0.001	0.429
Stimulation ^∗^ Group	6,88 = 3.21	6	0.015	0.180
Location	5,22 = 99.38	5	0.001	0.693
Location ^∗^ Group	15,22 = 1.97	15	0.058	0.118
Stimulation ^∗^ Location	10,44 = 13.63	10	0.001	0.236

**Between-subjects effects**	***F***		***P***	**$\eta_{p}^{2}$**

Group	3,44 = 2.5	3	0.072	0.146

**Table 3:** Mean values of stimulations across subject groups.

	HC (*n* = 16)	PD (*n* = 12)	PD w MCI (*n* = 10)	PDD (*n* = 10)
Stimulation	*M*(±*SE*)	*M*(±*SE*)	*M*(±*SE*)	*M*(±*SE*)
Target	7,43 ± 0.58	5,72 ± 0.66	5,53 ± 0.72	4,55 ± 0.72
Non-target	4,5 ± 0.3	3,98 ± 0.34	3,13 ± 0.37	3,45 ± 0.37
Simple stim	4,15 ± 0.35	4,28 ± 0.41	3,84 ± 0.44	4,30 ± 0.44

*M, mean; SE, standard error; HC, healthy controls; PD, Parkinson’s disease without cognitive deficits; PD w MCI, Parkinson’s disease with mild cognitive impairment; PDD, Parkinson’s disease with dementia.*

Stimulation effect was also significant [*F*_(df=2,88)_ = 33,1; *p* = 0.00; $\eta_{p}^{2}$ = 0.429], *post hoc* comparisons showed that target stimulation elicited higher delta responses than non-target (*p* < 0,0001) and simple auditory stimulation (*p* < 0,0001). Location effect was significant [*F*_(df=5,22)_ = 99.38; *p* = 0.001; $\eta_{p}^{2}$ = 0.693], *post hoc* comparisons showed that frontal locations had higher delta responses than temporal, temporal-parietal, parietal and occipital locations (*p* < 0,0001 for all comparisons). Delta responses over central locations were also higher than temporal, temporal-parietal, parietal and occipital locations (*p* < 0.0001 for all comparisons). There was a tendency for Location × group effect [*F*_(df=1,97)_ = 15,22; *p* = 0.058; $\eta_{p}^{2}$ = 0.118] **Table 4** represents the mean values of delta responses for locations separately for all groups of subjects. Although it did not reach to significant level there were differences between groups especially over frontal and central regions, healthy controls had higher delta responses over frontal and central areas in comparison to PD patients.

**Table 4:** Mean values of locations across subject groups.

	HC (*n* = 16)	PD (*n* = 12)	PD w MCI (*n* = 10)	PD w Dem (*n* = 10)
Location	*M*(±*SE*)	*M*(±*SE*)	*M*(±*SE*)	*M*(±*SE*)
Frontal (F_3_+F_4_)	7,67 ± 0.52	5,99 ± 0.60	5,99 ± 0.66	5,80 ± 0.66
Central (C_3_+C_4_)	7,04 ± 0.52	6,33 ± 0.59	5,61 ± 0.65	5,22 ± 0.65
Temporal (T_7_+T_8_)	3,67 ± 0.28	3,71 ± 0.32	3,12 ± 0.35	3,30 ± 0.35
Tempo-parietal (TP_7_+TP_8_)	3,82 ± 0.27	3,41 ± 0.30	2,79 ± 0.33	2,86 ± 0.33
Parietal (P_3_+P_4_)	5,93 ± 0.41	5,05 ± 0.47	4,29 ± 0.51	3,80 ± 0.51
Occipital (O_1_+O_2_)	4,03 ± 0.3	3,48 ± 0.34	3,19 ± 0.38	3,62 ± 0.38

*M, mean; SE, standard error; HC, healthy controls; PD: Parkinson’s disease without cognitive deficits; PD w MCI, Parkinson’s disease with mild cognitive impairment; PDD, Parkinson’s disease with dementia.*

Stimulation × location effect was significant [*F*_(df=10,44)_ = 13,63; *p* = 0.001; $\eta_{p}^{2}$ = 0.236], *post hoc* comparisons showed that target stimulation elicited higher delta responses than non-target and simple auditory stimulation especially at frontal and parietal locations (Frontal target vs.-non-target *p* < 0,0001; parietal target vs. non-target *p* < 0,0001; Frontal target vs.-simple auditory *p* < 0,0001; parietal target vs. simple auditory *p* < 0,0001).

**FIGURE 4:**
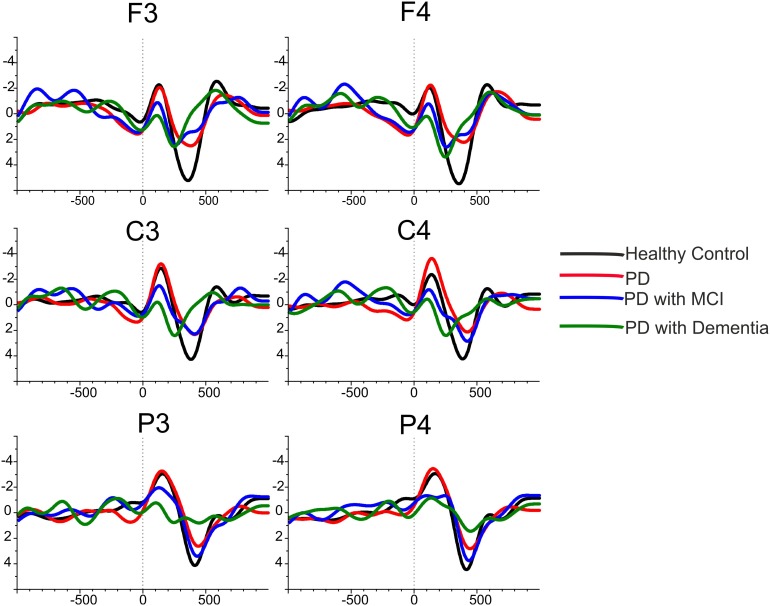
The grand average of delta responses upon application of target stimuli for healthy controls (black line), for Parkinson’s disease without cognitive deficit (red line), for Parkinson’s disease with MCI (blue line) and for Parkinson’s disease with dementia (green line).

Statistical results defined in the above paragraphs are also represented well in the grand averages of delta responses. The **Figure 4** illustrates the grand-average of delta responses upon application of target stimulation for healthy controls (black line, *N* = 16), for Parkinson’s disease without cognitive deficits (red line, *N* = 12), for PD with MCI (blue line, *N* = 10)and for PD with dementia (green line, *N* = 10) over frontal, central, and parietal locations. As is it can be seen in the figure healthy subjects had higher delta responses than the patient groups. As the cognitive deficit increased the delta response had decreased, Parkinson’s disease with dementia (green line) had the lowest delta response in comparison to other subject groups.

**FIGURE 5:**
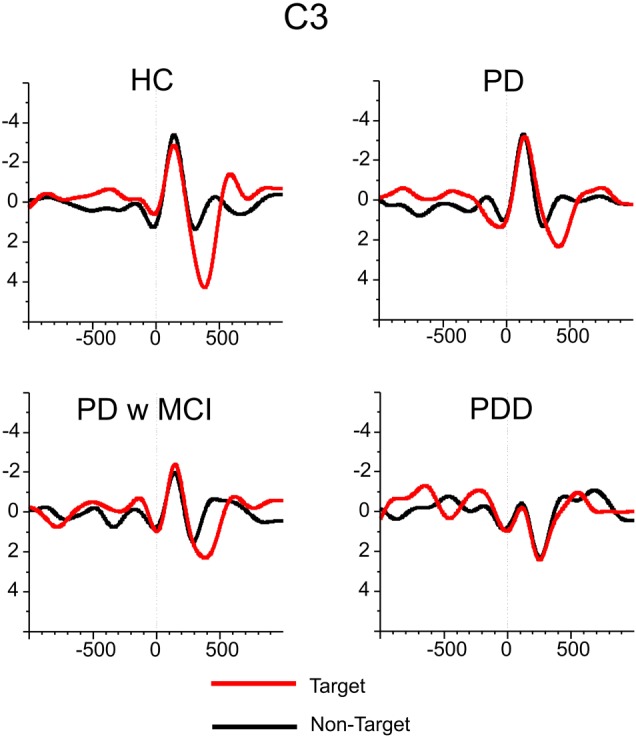
The grand averages of delta responses upon application of target (red line) and non-target (black line) stimulation for healthy controls, for Parkinson’s disease without cognitive deficit, for Parkinson’s disease with MCI and for Parkinson’s disease with dementia.

**Figure 5** illustrates the grand-average of delta responses upon application of target (red line) and non-target (black line) stimulation for all groups of subjects. As it can be seen in the figure, the healthy controls elicited higher delta responses during target stimulation in comparison to non-target stimulation. However, the difference between delta responses during target vs. non-target response decreased in patient groups. Delta responses during target stimulation were higher than non-target stimulation in PD patients without cognitive deficits and in PD patients with MCI, but the difference between target vs. non-target stimulation was not as much as the healthy controls. The most important observation of the present data was found for the PD patients with dementia. The difference between delta response during target stimulation vs. delta response during non-target stimulation diminished in patients with PD with dementia.

## Discussion

The present manuscript for the first time in the literature showed that delta responses gradually decrease according to the cognitive impairment in patients with Parkinson’s disease. In healthy controls, target stimulation elicited higher delta responses than non-target stimulation and simple auditory stimulation. Furthermore, during target stimulation, delta responses of healthy subjects were higher than all three group of the patients with PD (no cognitive decline, MCI, dementia). PD patients with dementia had the lowest amplitude in comparison to all other groups. There were no significant group differences for non-target simulation and simple auditory stimulation. Significant results were also found for UPDRS scores confirming previous research. PD patients with dementia had higher UPDRS scores than PD patients without cognitive deficits. Cognitive impairment in PD patients is related strongly to age and high Hoehn and Yahr scores (Aarsland et al., 2001; Verbaan et al., 2007). The patients who develop gait disorder and postural instability as the disease progresses, (these findings increase the UPDRS scores) are strong candidates for dementia (Alves et al., 2006).

### Event Related Potential Studies in Parkinson’s Disease

The decrease of P300 amplitude in PD patients was reported by several researchers (Antal et al., 1996; Pulvermuller et al., 1996; Philipova et al., 1997; Tsuchiya et al., 2000; Solís-Vivanco et al., 2011, 2015). Delayed P300a latencies were found in PD patients especially for the ones who had dementia (Goodin and Aminoff, 1986; Graham et al., 1990; Ebmeier et al., 1992; Tachibana et al., 1992). Mathis et al. (2014) and Kaufman et al. (2016) showed that apathy scores were correlated with a decrease of P300a amplitude. Solís-Vivanco et al. (2015) indicated that the reduced P300a amplitude in PD patients was related to the duration of PD and the severity of the illness. As the ERPs have amplitude and time characteristics, they also have frequency characteristics. P300 responses were reported to be the superposition of different frequency bands. Delta, theta, alpha, and gamma frequency bands were reported to shape P300 responses (Başar-Eroglu et al., 1991, 1993, 2000; Kolev et al., 1997; Schurmann et al., 1997; Demiralp et al., 1999, 2001; Spencer and Polich, 1999; Intriligator and Polich, 1994; Yordanova et al., 2000; Sakowitz et al., 2001; Güntekin et al., 2013). Many researcher showed that the major operating rhythms of P300 are mainly the delta and theta responses (Başar-Eroglu et al., 1992; Kolev et al., 1997; Demiralp et al., 1999, 2001; Spencer and Polich, 1999; Karakaş et al., 2000; Yordanova et al., 2000; Başar et al., 2001). The relation between alpha frequency and P300 was also studied (Intriligator and Polich, 1994; Spencer and Polich, 1999; Yordanova et al., 2001). Intriligator and Polich (1994) showed that low alpha (8–10 Hz) spectral power were correlated with P300 amplitude. Spencer and Polich (1999) found increased alpha-1 (7.5–9.5 Hz) and alpha-2 (9.5–12.5 Hz) power related to the increased task related attentional demands during an auditory oddball paradigm. Yordanova et al. (2001) reported that, event related alpha desynchronization increased as P300 latency became shorter.

Results of our study clearly showed that ERPs filtered in delta frequency band were impaired in PD patients. Healthy controls had increased delta responses during perception of target stimulation in comparison to non-target stimulation. However, the amplitude difference in delta response found between the target and non-target stimulations were not prominent in PD patients. The difference between target and non-target responses totally diminished in PD patients with dementia. Other frequency bands that shapes the ERPs should also be analyzed in future studies. We focused our attention to delta responses because delta responses were impaired in other cognitively impaired patient groups. In the hypothesis of our study we were expecting a decrease of delta response in PD patients, we have also hypothesized that PD patients with dementia would have the lowest delta responses in comparison to all other groups. Our previous results also showed similar results for MCI and AD patients. As the patients had more severe cognitive deficits delta responses decreased more (Yener and Başar, 2013).

### Event Oscillation Studies in Parkinson’s Disease

**Table 5** represents the event related oscillations studies performed by a different group of scientists. To our knowledge event related oscillatory studies in PD were few in comparison to spontaneous EEG and ERP studies. The research on this topic is still new, and it has to be enlarged in the coming years. As it is seen in **Table 5**, the researchers mostly analyzed theta and alpha responses during different cognitive stimulations. Schmiedt-Fehr et al. (2007) reported increased occipital delta responses in PD patients during Simon Task. Dushanova et al. (2009) reported that healthy controls had delta Event related synchronization in response to the low tone in 250–600 ms; on the other hand, PD patients had delta Event related desynchronization. The subjects who were included in these studies were cognitively normal PD patients. The number of PD patients in these studies was between 7 and 28 subjects. The present manuscript differentiates from other studies by comparing the sub-groups of PD patients. The present study included cognitively normal PD patients and as well PD patients with MCI and dementia. The present research for the first time in the literature showed that the event related delta responses were decreased gradually in PD patients according to their cognitive impairment. There are still few studies analyzing event related oscillations in PD. More research should be performed by considering different cognitive states in PD.

**Table 5:** Event related oscillation studies in Parkinson’s disease.

	PD group	Task	Frequency	Results
Schmiedt-Fehr et al., 2007	11 right non-demented mild to moderate PD	Simon task	Delta, theta	Increased Parietal-Occipital Delta, delayed delta and theta responses
Schmiedt et al., 2005	14 right non-demented mild to moderate PD	Shape tracking task	Theta, alpha	Less theta increase and upper alpha suppression
Huebl et al., 2014	Local field potential of subthalamic nucleus of 28 PD undergoing deep brain stimulation	IAPS (Emotional Pictures)	2–8 Hz, 8–12 Hz, 13–30 Hz	Early gamma band increase with unpleasant stimuli ON but not OFF medication; pleasant stimuli induced larger late alpha-ERD compared to neutral stimuli -ON medication
Ellfolk et al., 2006	7 mild stage PD patients	Sternberg’s memory search paradigm	Theta, alpha	Alpha ERS in posterior electrodes was observed in the controls, but not in the PD patients
Dushanova et al., 2009	16 PD patients	Auditory discrimination task	Delta, theta, Alpha	delta-ERS in CS and delta-ERD in PP in response to the low tone in 250–600 ms
Dushanova et al., 2010	16 PD patients	Auditory discrimination task	Beta, gamma	healthy controls had higher event related beta (13–20 Hz) synchronization than PD patients in a late time window
Emek-Savaş et al., 2017	16 PD patients	Visual Oddball Visual simple light	Delta	Decrease of delta responses
Present study	32 PD patients (12 PD, 10 PD-MCI and 10 PD-dementia)	Auditory Oddball Simple Auditory stimulation	Delta	Gradual decrease of delta responses during auditory oddball paradigm due to cognitive decline in PD

In the present study, the patients were on medication during the EEG recordings. Therefore it was not possible to control the effects of medication. The effect of L-Dopa on spontaneous EEG, ERPs (Georgiev et al., 2015) and as well as in different frequency bands were reported (Huebl et al., 2014). However, little is known about the effect of medication on event related oscillations in PD patients with different cognitive states, and it remains an essential question.

### What Does Decrease of Delta Response Mean?

In the present study, we have once more showed that delta responses increased during target stimulation in comparison non-target stimulation. This finding is a very robust finding of earlier literature (Başar and Stampfer, 1985; Yener et al., 2008, 2012; Güntekin and Başar, 2016). Furthermore, the results of the present study once more strengthen the hypothesis that the decrease of delta oscillatory responses is a general electrophysiological indicator for the cognitive impairment (Güntekin and Başar, 2016). Previous studies on delta responses of different patient groups showed that delta responses of patients with cognitive impairment had reduced delta responses upon cognitive load. Yener et al. (2008), showed reduced delta responses in AD patients both during visual (2008) and auditory (2012) oddball paradigm. Furthermore, a decrease of delta response during cognitive load found to be related to a loss in frontal volume in patients with MCI (Yener et al., 2016). The decrease of delta response was not just reported in dementia patients, but also in other patient groups with cognitive deficits. Schizophrenia patients (Röschke and Fell, 1997; Ergen et al., 2008; Ford et al., 2008) and patients with bipolar disorder (Atagün et al., 2014) also had decreased delta responses in comparison to healthy controls during cognitive stimulations.

Our group had previously analyzed delta responses in cognitively normal PD patients during visual oddball paradigm. That study by Emek-Savaş et al. (2017) had completely different patient and healthy control groups than the present study. Despite different patient groups Emek-Savaş et al. (2017) showed that cognitively normal PD patients had decreased delta responses than healthy controls during visual oddball paradigm. The present study for the first time showed that PD patients had also decreased delta responses during target stimulation of auditory oddball paradigm. Furthermore, the present study indicated a gradual decrease of delta responses in PD patients. As the PD patients had more cognitive decline delta responses decreased more. PD patients with dementia had the lowest delta response in comparison to all other groups especially during target stimulation (**Figure 3**). The decrease of delta response was only significant between groups during perception of target stimulation. There were no significant differences between groups during perception of non-target and simple auditory stimulation.

### Auditory Stimulation and Cognitive Decline in Parkinson’s Disease Patients

The visual system of PD patients was reported to be impaired by several scientists (Bodis-Wollner and Yahr, 1978; Bodis-wollner et al., 1987; Bodis-Wollner, 1990, 2003; Armstrong, 2011). Davidsdottir et al. (2005) reported that 78% of participants in their study endorsed at least one problem related to vision or visuospatial functioning. Regarding the perception deficits, PD patients had more severe deficits in the visual system and olfactory system (Metzler-Baddeley, 2007). It is also well known that PD patients show deficits in odor perception (Metzler-Baddeley, 2007). The auditory system of PD patients was known to be less affected than the visual and olfactory systems. Some studies indicated the positive role of dopaminergic treatment in the auditory paradigms. Georgiev et al. (2015) showed that the effect of medication on P3a response was observed only during auditory stimulations but not during visual stimulations. Geiser and Kaelin-Lang (2011) showed that auditory pulse perception was preserved in PD patients during the early stages of the disease. On the other hand, these authors also showed that PD patients displayed shorter reaction times after the administration of l-DOPA in comparison to before administration of l-DOPA. The present study showed that PD patients without cognitive deficits were as good as healthy controls in the identification of target simulation. However, the cognitive impairment affected the results importantly. Patients with PD MCI were worse than healthy controls and PD patients without any cognitive deficits. Most of the patients with dementia were not able to count targets correctly. All of our patients were on dopaminergic medication; they had their daily medication just 1 h before the EEG recordings. If we were able to collect data during on and off medication periods, we could also find differences between on and off conditions. The PD patients could be even worse if they were not on medication during the recordings. The effect of medication should also be analyzed in the future studies. One of the limitations our study was the lack of detailed medical auditory examination of the subject groups. In future, this examination should also be performed.

In the treatment of motor functions in PD patients, auditory stimulations were commonly used. Some researchers analyzed the effect of different sensory stimulations on gait in PD patients. Auditory stimulations have been reported to be more efficient than visual stimulations (Arias and Cudeiro, 2008). However, it is to note that PD patients could have difficulties in understanding the auditory stimulations when they have cognitive decline. The present study showed that as the cognitive decline increased the PD patients made more mistakes in identification of “target” stimulation. Most of the patients with dementia were not able to count targets correctly. On the other hand, PD patients without any cognitive impairment were as good as the healthy controls. The research on using the auditory stimulation in gait control in PD patients should also consider the cognitive functions of PD patients.

### Limitations of the Present Study

One of the important limitations of the study was the behavioral results of PD with dementia. This group subjects could not count the “target” stimulation as well as the other groups. Although this could be an expected result, we have also to consider that the paradigm applied to this group of subjects would not have the same effect as in other groups. In future more easier cognitive paradigms could be applied to PD patients with dementia. Patients with dementia were older in comparison to other groups (*p* = 0.072), this could also be seen as a limitation. On the other hand, it is also evident that there are very few subjects who had dementia and Parkinson’s disease in their early age. Accordingly, age difference could occur anytime when we want to compare the PD patients without cognitive deficits and PD patients with dementia.

## Conclusion

The present study for the first time in the literature showed that cognitive decline in Parkinson’s disease was represented with a gradual decrease of delta responses during auditory cognitive stimulation. There was significant group difference only during “target” stimulation. Increased cognitive function upon identification of “target” stimulation was represented with increased delta responses in healthy controls. However, PD patients with cognitive deficits had reduced delta responses. The group differences were not found during “non-target” stimulation or “simple auditory stimulation.” Therefore it can be assumed that the decrease delta response was related to cognitive decline. The present study once more strengthens the hypothesis that decrease of delta response is a general electrophysiological indicator of cognitive impairment. However, caution is also needed that the role of delta response could not be just considered as an indicator of cognitive decline. As we also mentioned in our recent review (Güntekin and Başar, 2016) delta response has a complex pre-stimulus and post-stimulus dynamics during different sensory and cognitive functions. Further research is needed to see how the decrease of delta response differentiates between different types of cognitive impairment. Since the effect of medication was reported on auditory perception in PD patients (Georgiev et al., 2015), in future, the effect of medication should also be studied.

## Author Contributions

BG, LH, and GY initiated the study and designed the protocol. BG wrote the paper. DG and TA recorded the EEG and analyzed the EEG data. DE-S helped to analyze the EEG data. NY, FÖ, and LH diagnosed the patients. FÇ and NM performed the neuropsychological tests and helped EEG recordings. EB supervised and controlled the study. All authors made a substantial contribution, drafted the manuscript, and approved the final manuscript.

## Conflict of Interest Statement

The authors declare that the research was conducted in the absence of any commercial or financial relationships that could be construed as a potential conflict of interest.

## References

[B1] Aarsland D. Andersen K. Larsen J. P. Lolk A. Kragh-Sørensen P. (2003). Prevalence and characteristics of dementia in Parkinson disease: an 8-year prospective study. *Arch. Neurol.* 60 387–392. 10.1001/archneur.60.3.387 12633150

[B2] Aarsland D. Andersen K. Larsen J. P. Lolk A. Nielsen H. Kragh–Sørensen P. (2001). Risk of dementia in Parkinson’s disease a community-based, prospective study. *Neurology* 56 730–736. 10.1212/WNL.56.6.730 11274306

[B3] Aarsland D. Brønnick K. Fladby T. (2011). Mild cognitive impairment in Parkinson’s disease. *Curr. Neurol. Neurosci. Rep.* 11 371–378. 10.1007/s11910-011-0203-1 21487730

[B4] Alves G. Larsen J. P. Emre M. Wentzel-Larsen T. Aarsland D. (2006). Changes in motor subtype and risk for incident dementia in Parkinson’s disease. *Mov. Disord.* 21 1123–1130. 10.1002/mds.20897 16637023

[B5] Antal A. Pfeiffer R. Bodis-Wollner I. (1996). Simultaneously evoked primary and cognitive visual evoked potentials distinguish younger and older patients with Parkinson’s disease. *J. Neural Transm.* 103 1053–1067. 10.1007/BF01291790 9013393

[B6] Aotsuka A. Weate S. J. Drake M. E. Jr. (1996). Event-related potentials in Parkinson’s disease. *Electromyogr. Clin. Neurophysiol.* 36 215–220. 8803493

[B7] Arias P. Cudeiro J. (2008). Effects of rhythmic sensory stimulation (auditory, visual) on gait in Parkinson’s disease patients. *Exp. Brain Res.* 186 589–601. 10.1007/s00221-007-1263-y 18214453

[B8] Armstrong R. A. (2011). Visual symptoms in Parkinson’s Disease. *Parkinsons Dis.* 2011:908306. 10.4061/2011/908306 21687773 PMC3109513

[B9] Atagün M. I. Güntekin B. Maşali B. Tülay E. Başar E. (2014). Decrease of event-related delta oscillations in euthymic patients with bipolar disorder. *Psychiatry Res.* 223 43–48. 10.1016/j.pscychresns.2014.04.001 24819306

[B10] Babiloni C. Del Percio C. Lizio R. Noce G. Cordone S. Lopez S. (2017). Abnormalities of cortical neural synchronization mechanisms in subjects with mild cognitive impairment due to Alzheimer’s and Parkinson’s Diseases: an EEG study. *J. Alzheimers Dis.* 59 339–358. 10.3233/JAD-160883 28621693

[B11] Başar E. Başar-Eroglu C. Karakaş S. Schürmann M. (2001). Gamma, alpha, delta, and theta oscillations govern cognitive processes. *Int. J. Psychophysiol.* 39 241–248. 10.1016/S0167-8760(00)00145-8 11163901

[B12] Başar E. Başar-Eroglu C. Rosen B. Schütt A. (1984). A new approach to endogenous event-related potentials in man: relation between EEG and P300-wave. *Int. J. Neurosci.* 24 1–21. 10.3109/00207458409079530 6480248

[B13] Başar E. Stampfer H. G. (1985). Important associations among EEG-dynamics, event-related potentials, short-term memory and learning. *Int. J. Neurosci.* 26 161–180. 10.3109/00207458508985615 4019045

[B14] Başar-Eroglu C. Başar E. (1991). A compound P300-40 Hz response of the cat hippocampus. *Int. J. Neurosci.* 60 227–237. 10.3109/00207459109080642 1787051

[B15] Başar-Eroglu C. Başar E. Demiralp T. Schürmann M. (1992). P300-response: possible psychophysiological correlates in delta and theta frequency channels. A review. *Int. J. Psychophysiol.* 13 161–179. 10.1016/0167-8760(92)90055-G 1399755

[B16] Başar-Eroglu C. Basar E. Schmielau F. (1991). P300 in freely moving cats with intracranial electrodes. *Int. J. Neurosci.* 60 215–226. 10.3109/00207459109080641 1787050

[B17] Başar-Eroglu C. Demiralp T. Schürmann M. Başar E. (2000). Topological distribution of oddball “P300” responses. *Int. J. Psychophysiol.* 39 213–220. 10.1016/S0167-8760(00)00142-2 11163898

[B18] Başar-Eroglu C. Strüber D. Stadler M. Kruse P. (1993). Multistable visual perception induces a slow positive EEG wave. *Int. J. Neurosci.* 73 139–151. 10.3109/00207459308987220 8132415

[B19] Bernat E. M. Malone S. M. Williams W. J. Patrick C. J. Iacono W. G. (2007). Decomposing delta, theta, and alpha time-frequency ERP activity from a visual oddball task using PCA. *Int. J. Psychophysiol.* 64 62–74. 10.1016/j.ijpsycho.2006.07.015 17027110 PMC2276568

[B20] Bodis-Wollner I. (1990). Visual deficits related to dopamine deficiency in experimental animals and Parkinson’s disease patients. *Trends Neurosci.* 13 296–302. 10.1016/0166-2236(90)90113-O 1695407

[B21] Bodis-Wollner I. (2003). Neuropsychological and perceptual defects in Parkinson’s disease. *Parkinsonism Relat. Disord.* 9(Suppl. 2), S83–S89. 10.1016/S1353-8020(03)00022-1 12915072

[B22] Bodis-wollner I. Marx M. S. Mitra S. Bobak P. Mylin L. Yahr M. (1987). Visual dysfunction in Parkinson’s disease: loss in spatiotemporal contrast sensitivity. *Brain* 110 1675–1698. 10.1093/brain/110.6.1675 3427405

[B23] Bodis-Wollner I. Yahr M. D. (1978). Measurements of visual evoked potentials in Parkinson’s disease. *Brain* 101 661–671. 10.1093/brain/101.4.661 737524

[B24] Bohnen N. I. Albin R. L. (2011). The cholinergic system and Parkinson disease. *Behav. Brain Res.* 221 564–573. 10.1016/j.bbr.2009.12.048 20060022 PMC2888997

[B25] Bonanni L. Thomas A. Tiraboschi P. Perfetti B. Varanese S. Onofrj M. (2008). EEG comparisons in early Alzheimer’s disease, dementia with Lewy bodies and Parkinson’s disease with dementia patients with a 2-year follow-up. *Brain* 131 690–705. 10.1093/brain/awm322 18202105

[B26] Brodaty H. Moore C. M. (1997). The clock drawing test for dementia of the Alzheimer’s type: a comparison of three scoring methods in a memory disorders clinic. *Int. J. Geriatr. Psychiatry* 12 619–627. 10.1002/(SICI)1099-1166(199706)12:6<619::AID-GPS554>3.0.CO;2-H 9215942

[B27] Buter T. C. Van Den Hout A. Matthews F. E. Larsen J. P. Brayne C. Aarsland D. (2008). Dementia and survival in Parkinson disease: a 12-year population study. *Neurology* 70 1017–1022. 10.1212/01.wnl.0000306632.43729.24 18362281

[B28] Caviness J. N. Driver-Dunckley E. Connor D. J. Sabbagh M. N. Hentz J. G. Noble B. (2007a). Defining mild cognitive impairment in Parkinson’s disease. *Mov. Disord.* 22 1272–1277. 10.1002/mds.21453 17415797

[B29] Caviness J. N. Hentz J. G. Evidente V. G. Driver-Dunckley E. Samanta J. Mahant P. (2007b). Both early and late cognitive dysfunction affects the electroencephalogram in Parkinson’s disease. *Parkinsonism Relat. Disord.* 13 348–354. 10.1016/j.parkreldis.2007.01.003 17347022

[B30] Chia L. G. Cheng L. J. Chuo L. J. Cheng F. C. Cu J. S. (1995). Studies of dementia, depression, electrophysiology and cerebrospinal fluid monoamine metabolites in patients with Parkinson’s disease. *J. Neurol. Sci.* 133 73–78. 10.1016/0022-510X(95)00146-S 8583235

[B31] Crawford P. (1992). “Assessment of frontal lobe dysfunction,” in *A Handbook of Neuropsychological Assessment*, eds Crawford J. R. Parker D. M. McKinlay W. W. (New York, NY: Erlbaum).

[B32] Daniel S. E. Lees A. J. (1993). Parkinson’s disease society brain bank, London: overview and research. *J. Neural. Transm.* 39 165–172. 8360656

[B33] Davidsdottir S. Cronin-Golomb A. Lee A. (2005). Visual and spatial symptoms in Parkinson’s disease. *Vis. Res.* 45 1285–1296. 10.1016/j.visres.2004.11.006 15733961

[B34] Demiralp T. Ademoglu A. Istefanopulos Y. Başar-Eroglu C. Başar E. (2001). Wavelet analysis of oddball P300. *Int. J. Psychophysiol.* 39 221–227. 10.1016/S0167-8760(00)00143-4 11163899

[B35] Demiralp T. Yordanova J. Kolev V. Ademoglu A. Devrim M. Samar V. J. (1999). Time-frequency analysis of single-sweep event-related potentials by means of fast wavelet transform. *Brain Lang.* 66 129–145. 10.1006/brln.1998.2028 10080868

[B36] Dushanova J. Philipova D. Nikolova G. (2009). Event-related desynchronization/synchronization during discrimination task conditions in patients with Parkinson’s disease. *Cell. Mol. Neurobiol.* 29 971–980. 10.1007/s10571-009-9384-4 19291392 PMC11505802

[B37] Dushanova J. Philipova D. Nikolova G. (2010). Beta and gamma frequency-range abnormalities in Parkinsonian patients under cognitive sensorimotor task. *J. Neurol. Sci.* 293 51–58. 10.1016/j.jns.2010.03.008 20392453

[B38] Ebmeier K. P. Potter D. D. Cochrane R. H. Crawford J. R. Stewart L. Calder S. A. (1992). Event related potentials, reaction time, and cognitive performance in idiopathic Parkinson’s disease. *Biol. Psychol.* 33 73–89. 10.1016/0301-0511(92)90007-H 1600001

[B39] Ellfolk U. Karrasch M. Laine M. Pesonen M. Krause C. M. (2006). Event-related desynchronization/synchronization during an auditory-verbal working memory task in mild Parkinson’s disease. *Clin. Neurophysiol.* 117 1737–1745. 10.1016/j.clinph.2006.05.004 16807091

[B40] Elwan O. H. Baradah O. H. Madkour O. Elwan H. Hassan A. A. H. Elwan F. (1996). Parkinson’s disease, cognition and aging. Clinical, neuropsychological, electrophysiological and cranial computerized tomographic assessment. *J. Neurol. Sci.* 143 64–71. 10.1016/S0022-510X(96)00161-X 8981300

[B41] Emek-Savaş D. D. Ozmus G. Guntekin B. Donmez Colakoglu B. Cakmur R. Basar E. (2017). Decrease of delta oscillatory responses in cognitively normal Parkinson’s disease. *Clin. EEG Neurosci.* 48 355–364. 10.1177/1550059416666718 27582502

[B42] Emre M. Aarsland D. Brown R. Burn D. J. Duyckaerts C. Mizuno Y. (2007). Clinical diagnostic criteria for dementia associated with Parkinson’s disease. *Mov. Disord.* 22 1689–1707. 10.1002/mds.21507 17542011

[B43] Ergen M. Marbach S. Brand A. Başar-Eroglu C. Demiralp T. (2008). P3 and delta band responses in visual oddball paradigm in schizophrenia. *Neurosci. Lett.* 440 304–308. 10.1016/j.neulet.2008.05.054 18571323

[B44] Fénelon G. Mahieux F. Huon R. Ziégler M. (2000). Hallucinations in Parkinson’s disease: prevalence, phenomenology and risk factors. *Brain* 123(Pt 4), 733–745. 10.1093/brain/123.4.733 10734005

[B45] Ford J. M. Roach B. J. Hoffman R. S. Mathalon D. H. (2008). The dependence of P300 amplitude on gamma synchrony breaks down in schizophrenia. *Brain Res.* 1235 133–142. 10.1016/j.brainres.2008.06.048 18621027 PMC3230270

[B46] Geiser E. Kaelin-Lang A. (2011). The function of dopaminergic neural signal transmission in auditory pulse perception: evidence from dopaminergic treatment in Parkinson’s patients. *Behav. Brain Res.* 225 270–275. 10.1016/j.bbr.2011.07.019 21787806

[B47] Georgiev D. Jahanshahi M. Dreo J. Čuš A. Pirtošek Z. Repovš G. (2015). Dopaminergic medication alters auditory distractor processing in Parkinson’s disease. *Acta Psychol.* 156 45–56. 10.1016/j.actpsy.2015.02.001 25697781

[B48] Gil R. Neau J. P. Toullat G. Rivasseau-Jonveaux T. Lefevre J. P. (1989). [Parkinson disease and cognitive evoked potentials]. *Rev. Neurol.* 145 201–207. 2749097

[B49] Goodin D. S. Aminoff M. J. (1986). Electrophysiological differences between subtypes of dementia. *Brain* 109 1103–1113. 10.1093/brain/109.6.1103 2947660

[B50] Graham J. S. Yiannikas C. Gordon E. Coyle S. Morris J. G. (1990). P300 event-related potentials in de novo Parkinson’s disease. *Clin. Exp. Neurol.* 27 89–98. 2129963

[B51] Green J. Woodard J. L. Sirockman B. E. Zakers G. O. Maier C. L. Green R. C. (1996). Event-related potential P3 change in mild Parkinson’s disease. *Mov. Disord.* 11 32–42. 10.1002/mds.870110108 8771065

[B52] Gungen C. Ertan T. Eker E. Yasar R. Engin F. (2002). [Reliability and validity of the standardized mini mental state examination in the diagnosis of mild dementia in Turkish population]. *Turk. Psikiyatri Derg.* 13 273–281. 12794644

[B53] Güntekin B. Başar E. (2014). A review of brain oscillations in perception of faces and emotional pictures. *Neuropsychologia* 58 33–51. 10.1016/j.neuropsychologia.2014.03.014 24709570

[B54] Güntekin B. Başar E. (2016). Review of evoked and event-related delta responses in the human brain. *Int. J. Psychophysiol.* 103 43–52. 10.1016/j.ijpsycho.2015.02.001 25660301

[B55] Güntekin B. Emek-Savaş D. D. Kurt P. Yener G. G. Başar E. (2013). Beta oscillatory responses in healthy subjects and subjects with mild cognitive impairment. *Neuroimage Clin.* 3 39–46. 10.1016/j.nicl.2013.07.003 24179847 PMC3791295

[B56] Hansch E. C. Syndulko K. Cohen S. N. Goldberg Z. I. Potvin A. R. Tourtellotte W. W. (1982). Cognition in Parkinson disease: an event-related potential perspective. *Ann. Neurol.* 11 599–607. 10.1002/ana.410110608 7114809

[B57] Hautecoeur P. Gallois P. Forzy G. Chatelet P. Choteau P. Dereux J. F. (1991). [Late auditory evoked potentials in subcortical cognitive deterioration]. *Rev. Neurol.* 147 293–299. 2063079

[B58] Hoehn M. M. Yahr M. D. (1967). Parkinsonism: onset, progression, and mortality. *Neurology* 57(10 Suppl. 3), S11–S26. 10.1212/WNL.17.5.427 11775596

[B59] Huebl J. Spitzer B. Brücke C. Schönecker T. Kupsch A. Alesch F. (2014). Oscillatory subthalamic nucleus activity is modulated by dopamine during emotional processing in Parkinson’s disease. *Cortex* 60 69–81. 10.1016/j.cortex.2014.02.019 24713195

[B60] Hughes T. A. Ross H. F. Musa S. Bhattacherjee S. Nathan R. N. Mindham R. H. S. (2000). A 10-year study of the incidence of and factors predicting dementia in Parkinson’s disease. *Neurology* 54 1596–1603. 10.1212/WNL.54.8.1596 10762499

[B61] Intriligator J. Polich J. (1994). On the relationship between background EEG and the P300 event-related potential. *Biol. Psychol.* 37 207–218. 10.1016/0301-0511(94)90003-5 7948466

[B62] Jiang C. Kaseda Y. Kumagai R. Nakano Y. Nakamura S. (2000). Habituation of event-related potentials in patients with Parkinson’s disease. *Physiol. Behav.* 68 741–747. 10.1016/S0031-9384(99)00244-9 10764905

[B63] Karakaş S. Erzengin O. U. Basar E. (2000). A new strategy involving multiple cognitive paradigms demonstrates that ERP components are determined by the superposition of oscillatory responses. *Clin. Neurophysiol.* 111 1719–1732. 10.1016/S1388-2457(00)00418-1 11018485

[B64] Karkas S. (2006). *BÝLNOT Bataryasí El Kitabí.* Ankara: Eryílmaz Ofset.

[B65] Kaufman D. A. S. Bowers D. Okun M. S. Van Patten R. Perlstein W. M. (2016). Apathy, novelty processing, and the P3 Potential in Parkinson’s disease. *Front. Neurol.* 7:95 10.3389/fneur.2016.00095 PMC4917554 27445962

[B66] Kehagia A. A. Barker R. A. Robbins T. W. (2010). Neuropsychological and clinical heterogeneity of cognitive impairment and dementia in patients with Parkinson’s disease. *Lancet Neurol.* 9 1200–1213. 10.1016/S1474-4422(10)70212-X 20880750

[B67] Kim G. W. Sohn Y. H. Huh K. Kim J. S. (1995). Relationship between the auditory P300 and the procedural memory function in drug-naive patients with Parkinson’s disease. *Yonsei Med. J.* 36 367–371. 10.3349/ymj.1995.36.4.367 7483680

[B68] Knyazev G. G. (2012). EEG delta oscillations as a correlate of basic homeostatic and motivational processes. *Neurosci. Biobehav. Rev.* 36 677–695. 10.1016/j.neubiorev.2011.10.002 22020231

[B69] Kolev V. Demiralp T. Yordanova J. Ademoglu A. Isoglu-Alkaç U. (1997). Time-frequency analysis reveals multiple functional components during oddball P300. *Neuroreport* 8 2061–2065. 10.1097/00001756-199705260-00050 9223102

[B70] Kurt P. Emek-SavaG D. D. Batum K. Turp B. Güntekin B. Karsidal S. (2014). Patients with mild cognitive impairment display reduced auditory event-related delta oscillatory responses. *Behav. Neurol.* 2014:268967. 10.1155/2014/268967 24825953 PMC4006610

[B71] Lang A. E. T. Fahn S. (1989). “Assessment of Parkinson’s disease,” in *Quantification of Neurologic Deficit*, ed. Munsat T. L. (Boston, MA: Butterworth).

[B72] Levy G. Tang M. X. Cote L. J. Louis E. D. Alfaro B. Mejia H. (2000). Motor impairment in PD: relationship to incident dementia and age. *Neurology* 55 539–544. 10.1212/WNL.55.4.539 10953188

[B73] Levy G. Tang M.-X. Louis E. D. Côté L. J. Alfaro B. Mejia H. (2002). The association of incident dementia with mortality in PD. *Neurology* 59 1708–1713. 10.1212/WNL.61.3.424 12473757

[B74] Lewis S. J. G. Dove A. Robbins T. W. Barker R. A. Owen A. M. (2003). Cognitive impairments in early Parkinson’s disease are accompanied by reductions in activity in frontostriatal neural circuitry. *J. Neurosci.* 23 6351–6356. 12867520 10.1523/JNEUROSCI.23-15-06351.2003 PMC6740550

[B75] Lin C. H. Wu R. M. (2015). Biomarkers of cognitive decline in Parkinson’s disease. *Parkinsonism Relat. Disord.* 21 431–443. 10.1016/j.parkreldis.2015.02.010 25737398

[B76] Litvan I. Goldman J. G. Tröster A. I. Schmand B. A. Weintraub D. Petersen R. C. (2012). Diagnostic criteria for mild cognitive impairment in Parkinson’s disease: movement disorder society task force guidelines. *Mov. Disord.* 27 349–356. 10.1002/mds.24893 22275317 PMC3641655

[B77] Mathes B. Schmiedt J. Schmiedt-Fehr C. Pantelis C. Basar-Eroglu C. (2012). New rather than old? For working memory tasks with abstract patterns the P3 and the single-trial delta response are larger for modified than identical probe stimuli. *Psychophysiology* 49 920–932. 10.1111/j.1469-8986.2012.01372.x 22524263

[B78] Mathis S. Neau J. P. Pluchon C. Fargeau M. N. Karolewicz S. Iljicsov A. (2014). Apathy in parkinson’s disease: an electrophysiological study. *Neurol. Res. Int.* 2014:290513. 10.1155/2014/290513 24804097 PMC3996982

[B79] Metzler-Baddeley C. (2007). A review of cognitive impairments in dementia with Lewy bodies relative to Alzheimer’s disease and Parkinson’s disease with dementia. *Cortex* 43 583–600. 10.1016/S0010-9452(08)70489-1 17715794

[B80] Morris J. C. (1993). The clinical dementia rating (CDR): current version and scoring rules. *Neurology* 43 2412–2414. 10.1212/WNL.43.11.2412-a 8232972

[B81] Moustafa A. A. Poletti M. (2013). Neural and behavioral substrates of subtypes of Parkinson’s disease. *Front. Syst. Neurosci.* 7:117 10.3389/fnsys.2013.00117 PMC3872046 24399940

[B82] Neufeld M. Y. Blumen S. Aitkin I. Parmet Y. Korczyn A. D. (1994). EEG frequency analysis in demented and nondemented parkinsonian patients. *Dementia* 5 23–28. 10.1159/000106690 8156083

[B83] Neufeld M. Y. Inzelberg R. Korczyn A. D. (1988). EEG in demented and non-demented parkinsonian patients. *Acta Neurol. Scand.* 78 1–5. 10.1111/j.1600-0404.1988.tb03609.x 3176875

[B84] O’Donnell B. F. Squires N. K. Martz M. J. Chen J. R. Phay A. J. (1987). Evoked potential changes and neuropsychological performance in Parkinson’s disease. *Biol. Psychol.* 24 23–37. 10.1016/0301-0511(87)90097-4 3567267

[B85] Öktem Ö. (2011). *Öktem Sözel Bellek Süreçleri Testi (Öktem SBST) El Kitabí.* Ankara: Türk Psikologlar Derneği Yayínlarí.

[B86] Philipova D. Gatchev G. Vladova T. Georgiev D. (1997). Event-related potentials in Parkinsonian patients under auditory discrimination tasks. *Int. J. Psychophysiol.* 27 69–78. 10.1016/S0167-8760(97)00783-6 9161893

[B87] Pollux P. M. J. (2004). Advance preparation of set-switches in Parkinson’s disease. *Neuropsychologia* 42 912–919. 10.1016/j.neuropsychologia.2003.12.002 14998705

[B88] Prada L. Barcel F. Herrmann C. S. Escera C. (2014). EEG delta oscillations index inhibitory control of contextual novelty to both irrelevant distracters and relevant task-switch cues. *Psychophysiology* 51 658–672. 10.1111/psyp.12210 24673586

[B89] Pugnetti L. Baglio F. Farina E. Alberoni M. Calabrese E. Gambini A. (2010). EEG evidence of posterior cortical disconnection in PD and related dementias. *Int. J. Neurosci.* 120 88–98. 10.3109/00207450903436346 20199199

[B90] Pulvermuller F. Lutzenberger W. Muller V. Mohr B. Dichgans J. Birbaumer N. (1996). P3 and contingent negative variation in Parkinson’s disease. *Electroencephalogr. Clin. Neurophysiol.* 98 456–467. 10.1016/0013-4694(96)95537-6 8763505

[B91] Röschke J. Fell J. (1997). Spectral analysis of P300 generation in depression and schizophrenia. *Neuropsychobiology* 35 108–114. 10.1159/000119400 9097303

[B92] Sakowitz O. W. Quiroga R. Q. Schürmann M. Başar E. (2001). Bisensory stimulation increases gamma-responses over multiple cortical regions. *Cogn. Brain Res.* 11 267–279. 10.1016/S0926-6410(00)00081-1 11275488

[B93] Schmiedt C. Meistrowitz A. Schwendemann G. Herrmann M. Basar-Eroglu C. (2005). Theta and alpha oscillations reflect differences in memory strategy and visual discrimination performance in patients with Parkinson’s disease. *Neurosci. Lett.* 388 138–143. 10.1016/j.neulet.2005.06.049 16040192

[B94] Schmiedt-Fehr C. Schwendemann G. Herrmann M. Basar-Eroglu C. (2007). Parkinson’s disease and age-related alterations in brain oscillations during a Simon task. *Neuroreport* 18 277–281. 10.1097/WNR.0b013e32801421e3 17314671

[B95] Schurmann M. Basar-Eroglu C. Basar E. (1997). Gamma responses in the EEG: elementary signals with multiple functional correlates. *Neuroreport* 8 1793–1796. 10.1097/00001756-199705060-00045 9189935

[B96] Seer C. Lange F. Georgiev D. Jahanshahi M. Kopp B. (2016). Event-related potentials and cognition in Parkinson’s disease: an integrative review. *Neurosci. Biobehav. Rev.* 71 691–714. 10.1016/j.neubiorev.2016.08.003 27498083

[B97] Serizawa K. Kamei S. Morita A. Hara M. Mizutani T. Yoshihashi H. (2008). Comparison of quantitative EEGs between Parkinson disease and age-adjusted normal controls. *J. Clin. Neurophysiol.* 25 361–366. 10.1097/WNP.0b013e31818f50de 18997624

[B98] Solís-Vivanco R. Ricardo-Garcell J. Rodríguez-Camacho M. Prado-Alcalá R. A. Rodríguez U. Rodríguez-Violante M. (2011). Involuntary attention impairment in early Parkinson’s disease: an event-related potential study. *Neurosci. Lett.* 495 144–149. 10.1016/j.neulet.2011.03.058 21443924

[B99] Solís-Vivanco R. Rodríguez-Violante M. Rodríguez-Agudelo Y. Schilmann A. Rodríguez-Ortiz U. Ricardo-Garcell J. (2015). The P3a wave: a reliable neurophysiological measure of Parkinson’s disease duration and severity. *Clin. Neurophysiol.* 126 2142–2149. 10.1016/j.clinph.2014.12.024 25655938

[B100] Soliveri P. Monza D. Paridi D. Carella F. Genitrini S. Testa D. (2000). Neuropsychological follow up in patients with Parkinson’s disease, striatonigral degeneration-type multisystem atrophy, and progressive supranuclear palsy. *J. Neurol. Neurosurg. Psychiatry* 69 313–318. 10.1136/jnnp.69.3.313 10945805 PMC1737110

[B101] Spencer K. M. Polich J. (1999). Poststimulus EEG spectral analysis and P300: attention, task, and probability. *Psychophysiology* 36 220–232. 10.1111/1469-8986.3620220 10194969

[B102] Tachibana H. Toda K. Sugita M. (1992). Actively and passively evoked P3 latency of event-related potentials in Parkinson’s disease. *J. Neurol. Sci.* 111 134–142. 10.1016/0022-510X(92)90061-O 1431980

[B103] Tanaka H. Koenig T. Pascual-Marqui R. D. Hirata K. Kochi K. Lehmann D. (2000). Event-Related Potential and EEG Measures in Parkinson’s Disease without and with Dementia. *Dement. Geriatr. Cogn. Disord.* 11 39–45. 10.1159/000017212 10629361

[B104] Tsuchiya H. Yamaguchi S. Kobayashi S. (2000). Impaired novelty detection and frontal lobe dysfunction in Parkinson’s disease. *Neuropsychologia* 38 645–654. 10.1016/S0028-3932(99)00108-6 10689041

[B105] Verbaan D. Marinus J. Visser M. van Rooden S. M. Stiggelbout A. M. Middelkoop H. A. (2007). Cognitive impairment in Parkinson’s disease. *J. Neurol. Neurosurg. Psychiatry* 78 1182–1187. 10.1136/jnnp.2006.112367 17442759 PMC2117586

[B106] Wechsler D. (1987). *Manual for Wechsler Memory Scale – Revised.* San Antonio, TX: Psychol. Corp.

[B107] Yener G. Güntekin B. Başar E. (2008). Event-related delta oscillatory responses of Alzheimer patients. *Eur. J. Neurol.* 15 540–547. 10.1111/j.1468-1331.2008.02100.x 18410376

[B108] Yener G. G. Başar E. (2013). Biomarkers in Alzheimer’s disease with a special emphasis on event-related oscillatory responses. *Suppl. Clin. Neurophysiol.* 62 237–273. 10.1016/B978-0-7020-5307-8.00020-X 24053044

[B109] Yener G. G. Emek-Savaş D. D. Lizio R. Çavuşoğlu B. Carducci F. Ada E. (2016). Frontal delta event-related oscillations relate to frontal volume in mild cognitive impairment and healthy controls. *Int. J. Psychophysiol.* 103 110–117. 10.1016/j.ijpsycho.2015.02.005 25660300

[B110] Yener G. G. Güntekin B. Örken D. N. Tülay E. Forta H. Başar E. (2012). Auditory delta event-related oscillatory responses are decreased in Alzheimer’s disease. *Behav. Neurol.* 25 3–11. 10.3233/BEN-2012-0344 22207418 PMC5294260

[B111] Yener G. G. Kurt P. Emek-Savaş D. D. Güntekin B. Başar E. (2013). Reduced visual event related δ oscillatory responses in amnestic mild cognitive impairment. *J. Alzheimers Dis.* 37 759–767. 10.3233/JAD-130569 23948923

[B112] Yordanova J. Devrim M. Kolev V. Ademoglu A. Demiralp T. (2000). Multiple time-frequency components account for the complex functional reactivity of P300. *Neuroreport* 11 1097–1103. 10.1097/00001756-200004070-00038 10790889

[B113] Yordanova J. Kolev V. Polich J. (2001). P300 and alpha event-related desynchronization (ERD). *Psychophysiology* 38 143–152. 10.1111/1469-8986.3810143 11321615

